# The Role of Hyaluronic Acid in Sport-Related Tendinopathies: A Narrative Review

**DOI:** 10.3390/medicina57101088

**Published:** 2021-10-12

**Authors:** Sergio Crimaldi, Sara Liguori, Pasquale Tamburrino, Antimo Moretti, Marco Paoletta, Giuseppe Toro, Giovanni Iolascon

**Affiliations:** 1 Humanitas Clinical and Research Center—IRCCS, 20900 Milan, Italy; sergiocrimaldi@gmail.com; 2 Department of Medical and Surgical Specialties and Dentistry, University of Campania “Luigi Vanvitelli”, 80100 Naples, Italy; antimo.moretti@unicampania.it (A.M.); marco.paoletta@unicampania.it (M.P.); giuseppe.toro@unicampania.it (G.T.); giovanni.iolascon@unicampania.it (G.I.); 3 Azienda USL Frosinone—UOC Ortopedia e Traumatologia, 03100 Frosinone, Italy; lellotamb@alice.it

**Keywords:** tendinopathy, hyaluronic acid, athletes, return to sport, conservative treatment

## Abstract

Tendinopathy is a complex clinical condition with a rising incidence and prevalence, particularly during sports practice. For the return to play in affected patients, adequate functional and structural recovery of the tendon is the ultimate goal, avoiding the high risk of recurrence. In this perspective, local therapies alongside exercise are showing promising results. Despite evidence suggesting hyaluronic acid (HA) injections as effective in the treatment of tendinopathy, current recommendations about the management of this condition do not include this intervention. HA seems to be an effective therapeutic option for the management of sport-related tendinopathies, but further studies with a larger sample size are needed to confirm available findings. In this narrative review, we analyzed available literature about the rationale of the use of HA in the management of tendon injury and, particularly, in sport-related tendinopathies.

## 1. Introduction

Tendinopathy is an umbrella term used to identify a complex clinical condition characterized by molecular, cellular, and histological changes occurring in affected tendons that leads to persistent pain, swelling, and impaired physical performance [1,2]. Several pathogenic mechanisms are involved in the occurrence of this condition. High load demands and repetitive mechanical exposure during exercise act as primum movens of tendinopathy. A persistent failed healing response leads to the progressive accumulation of matrix damage with microruptures of collagen fibrils in tendons. Moreover, tendon injury triggers the release of cytokines, chemokines, and other inflammatory molecules, responsible of the symptoms reported by affected patients [1].

Both prevalence and incidence of tendinopathy are increasing worldwide in the last decades, particularly in sport practice, where accounts for up to ~30% of total injuries [3]. In the general population, common sites of tendinopathy involve rotator cuff and extensor carpi radialis brevis tendons for the upper limb and gluteal, patellar, and Achilles’ tendons for the lower limb [4]. On the other side, in athletes, Achilles’ tendinopathy affects up to 30% of all runners, while patellar tendinopathy mostly affects volleyball and basketball player, with an incidence of 14% and 12%, respectively [5]. Several risk factors contribute to the development of tendinopathy including genetic susceptibility (i.e., polymorphisms in collagen type V alpha 1 chain- COL5A1, Tenascin C-TNC, Matrix Metallopeptidase 3-MMP3 and Estrogen Related Receptor Alpha- ESRRA), chronic diseases (i.e., diabetes), or specific pharmacological therapies (i.e., quinolone antibiotics) [1].

Recovery of an adequate tendon load-bearing capacity is mandatory among athletes, particularly due to the high risk of recurrence of tendinopathy observed after early return to sport [6]. Currently, recommended treatment strategies are extremely variable [1]. Exercise remains the most effective approach [7,8] and may be associated with the use of the local therapies as adjunctive treatment [2]. For several years, glucocorticoids (GC) have been used as the main injection treatment in patients with tendinopathy. Despite their large use in this population, the safety of GC is still debated [9,10,11]. Indeed, GC alter tendon homeostasis by inducing collagen disorganization and necrosis. Moreover, GC reduce tenocytes’ viability, promoting the cell senescence and consequently tendon ruptures [9,12].

Therefore, the interest of researchers and clinicians in investigating the role of other interventions for treating tendinopathy is growing. In particular, injections of hyaluronic acid (HA) have showed promising results in the treatment of this condition, particularly for sport-related tendinopathies [13,14]. This paper aims to describe the rationale of the HA administration in the management of tendinopathy and clinical implications in sport-related tendinopathies.

## 2. The Rationale of HA Use in the Management of Tendinopathies

Hyaluronic acid, also known as hyaluronan or hyaluronate, is a fascinating biologically active molecule, composed of repeating disaccharides of β-1,4-D-glucuronic acid, and β-1,3-N-acetylglucosamine units [15]. This compound is largely present in the extracellular matrix (ECM) of articular cartilage and in synovial fluid and is secreted by synovial cells of the tendon sheath. Rheological characteristics of HA contribute to lubrication, viscoelasticity, and hydration balance, acting as a shock absorber and a structure stabilizer in different tissues, including tendons [16]. Furthermore, HA seems to be involved in the regulation of the tissue repair process, modulating the main phases of tendon healing (i.e., inflammation, cellular migration, and angiogenesis) [17]. All these properties supported HA use as a conservative treatment for several musculoskeletal disorders, including tendinopathy [18,19]. 

### 2.1. Preclinical Studies

**Figure 1:**
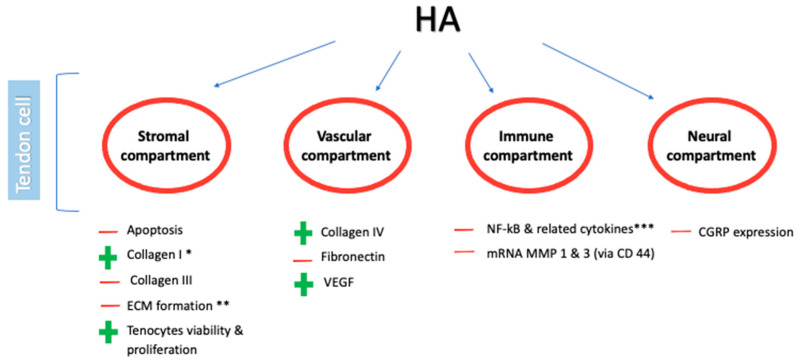
Biological effects modulated by HA (hyaluronic acid) on tendon cells. Note: − and + indicate negative and positive modulation, respectively. * dose-dependent, ** high concentration and HW, *** IL-1 α, IL-6, TNF-α. Abbreviations: ECM (extracellular matrix); VEGF (Vascular-Endothelial Growth Factor); MMP (matrix metalloproteinases); CGRP (Calcitonin Gene Related Peptide); HW (high weight).

In recent years, the potential effects of HA on tendon cells increased research interest. Indeed, HA seems to modulate several biological pathways involved in tendinopathy, with substantial benefits on tendon biomechanics [20,21,22,23] (Figure 1).

Hyaluronic acid improves tendon gliding limiting tendon adhesion in physiological condition [24,25]. 

In a preclinical study, Nishida et al. demonstrated in a model of canine flexor tendon graft, that 10 mg/mL HA administration significantly reduces the excursion resistance between the tendon and pulley [25].

Oryan et al. showed that, HA injection in tenotomized superficial digital flexor tendon in rabbits combined with oral administration of glucosamine HCl-chondroitin sulfate have beneficial effects in terms of differentiation, maturation, and alignment of the collagen fibrils, enhancing the biomechanical properties of the tendon compared to administration of saline injection and oral placebo [26]. 

Moreover, Kurt et al. compared HA versus synovial fluid (SF) for anti-adhesive purposes in a rabbit Achilles’ tendon model. The authors demonstrated that HA reduces tendon adhesion with a higher grade of tendon healing and mobility compared to SF group [27]. 

Hyaluronic acid seems to modulate inflammation through different pathways, including the suppression of proinflammatory molecules, as investigated by Wu et al. The authors demonstrated that HMW (high molecular weight) HA injections in IL-1β-stimulated tenocytes reduce mRNA and protein expression of matrix metalloproteinase (MMP)-1 and -3, overexpressed in inflamed tendons, in a dose-dependent manner. Moreover, the authors found that this intervention significantly reduces MMP-1 and -3 expression and pain (Visual Analogue Scale, VAS) also in patients with peritendinous effusion of long head of biceps one-month post-treatment [28]. 

In a murine model, Neumann et al. hypothesized that HMW-HA inhibits the inflammation signaling pathway (NF-kB) and NF-kB-regulated cytokines release (interleukin-1α, IL-1α, interleukin-6 IL-6, and tumor necrosis factor-α, TNF-α) caused by advanced glycation end products (AGEs) while LMW (low molecular weight) HA resulted in activation of NF-kB [20]. 

Hyaluronic acid seems also to enhance tenocytes’ viability as demonstrated by Yoshida et al. in a rat model of patellar tendinopathy. In this study the number and length of tendon’s microtears and laminations as well as the number of apoptotic tendon cells were significantly lower after HMW HA injections compared to saline injections or nothing (control groups) [29]. In an in vitro study, Gallorini et al. evaluated the effects of two different molecular weight HA (80–100 kDa and 800–1200 kDa, respectively) on human tendon cells derived from patients with rotator cuff tears. The authors demonstrated that both type of HA induce tenocytes’ proliferation after 72 h of exposure and decrease apoptotic cells population compared to the controls (not exposed to HA) [30]. In two groups of rabbits (16 per group) with surgical Achilles’ tendon wound, Halici et al. administered 0.5 mL of sodium hyaluronate (NaHA, 15 mg/mL 1.5 kDa) and 0.5 mL of saline solutions (two injections at one-week intervals for both the interventions), respectively. At the macroscopic evaluation, the authors observed significant increases in vascular endothelial growth factor (VEGF) and type IV collagen (a main component of the vascular basement membrane), after 6 week and a decrease in adhesion between the tendon and surrounding tissue in the NaHA group compared to the control group at 6 and 12 weeks [23]. These findings suggest that HA accelerates tendon repair by inducing the restoration process, suppressing apoptosis, and modulating angiogenesis. 

Finally, Yamaguchi et al. demonstrated that both HA and GC injections compared to saline injections lead to a suppression of inflammation through the reduction of the calcitonin gene-related peptide (CGRP) expression and provide functional improvements evaluated with gait analysis, in a rat rotator cuff tears (RCT) model [21]. On the other hand, in in vitro observation and in a similar animal model, Nakamura et al. showed that GC inhibits fibroblast proliferation and delays tendon healing, suggesting that GC interferes with the early phases of tendon repair, regeneration and remodeling compared to HA [31]. 

### 2.2. Clinical Studies

Several studies showed promising results about HA administration for treating rotator cuff (RC) tendinopathies [32,33,34]. Meloni et al. enrolled 56 patients with supraspinatus tendinopathy comparing periarticular injections of sodium HA (SH) and sodium chloride (SC), administrated at the baseline and once a week for 4 weeks (a total of five injections). The SH group improved pain score (VAS) after 4 weeks from the last injection and maintained the benefits up to 1 year [35].

In 48 participants with a clinical diagnosis of RC tendinopathy, Merolla et al. investigated the effectiveness of HA injections once weekly for 2 weeks versus 30 days of physiotherapy (25 and 23 participants per group, respectively). In the HA group, a meaningful reduction of pain was observed until the 12 weeks, while in the physiotherapy group a decrease in pain was reported only at week 2 with higher pain scores at 4, 12, and 24 weeks. A similar trend was observed for the clinical outcomes analyzed (Constant–Murley Score and Oxford Shoulder Score) [36].

In a double-blinded study, Petrella et al. compared two injections (once a week) of 1% SH versus saline injections in 331 competitive racquet sport athletes with lateral elbow pain of greater than 3 months. Statistically significant improvements in pain at rest and after maximal grip testing, grip strength, patient global satisfaction, and elbow function were found in the HA group, with a persistent between-group differences up to 1-year follow-up [13].

Hyaluronic acid plus chondroitin sulfate (CS) demonstrated some benefits on pain control in lateral epicondylitis in comparison with GC, as investigated by Tosun et al. in a prospective randomized study. Twenty-five patients received a single injection of HA plus CS while 32 patients were treated with a single injection of triamcinolone and prilocaine HCl. Although after 6 months both groups improved mean pain and function scores, the HA plus CS group had statistically significantly better scores in pain and function outcomes compared to triamcinolone. According to some studies, CS would contribute to the tendon healing [37,38], and seems to help HA in the maturation process of tenoblasts and tenocytes [39].

For what concern lower limb tendon injuries, Muneta et al. investigated the effectiveness and safety of LMW HA injection in 50 athletes (54% semiprofessional, 22% competitive, and 24% recreational sports) with patellar tendinopathy treated from January 1999 to December 2006 with an average of 2 injections per case. Based on subjective pain (during sporting activities) combined with recovery (ability to participate in athletic activities), results ranged from excellent (54%) to good (40%) and all of them were considered able to return to their previous sporting activities, according to modified Roles and Maudsley score [14]. 

Lynen et al. in a prospective, randomized study compared the efficacy in terms of pain of 2 peritendinous injections of HA versus 3 extracorporeal shock wave therapy (ESWT) sessions (both treatments at weekly intervals) in 29 and 30 participants with Achilles’ tendinopathy, respectively. Statistically significant improvements in VAS score were observed in the HA group at every follow-up (4 weeks: HA −68.1% vs. ESWT −47.9%, 3 months: HA −88.2% vs. ESWT −51.6%, 6 months: HA −94.9% vs. ESWT −66.4%). Moreover, 12.9% of participants experienced nonserious adverse events (3 patients in the HA group and 5 in the ESWT-group) [40].

More recently, Gervasi et al. investigated the effectiveness of three peritendinous injections of LMW HA in the treatment of unilateral AT in 8 middle-aged male runners examining visco-elastometric, biochemical, functional and clinical parameters. At 30 days after the first injection, the authors observed a reduction of inflammatory markers level (IL-1β and MMP-3). Pain relief, improvement of maximal voluntary isometric contraction (MVIC) and stiffness were significantly reached since the first injection and maintained over time in the injured tendon. Moreover, these results were significantly different to the healthy tendon (*p* = 0.028; *p* = 0.023; *p* = 0.047, respectively) [41].

Further details about clinical studies included in this paper are reported in Table 1.

## 3. Clinical Implications of HA Injections in Sport-Related Tendinopathies

Tendinopathies may occur in both elite and recreational athletes, with the contribution of several factors including intrinsic (i.e., reduced muscle flexibility) and extrinsic (i.e., increased training volume, eccentric exercises, hard surfaces) conditions [42]. The pathophysiological changes of tendon injured are well known, encompassing neurovascular in-growth, abnormal tendon density and degeneration of ECM, all induced by overuse and high load demand of tendon [43]. Nevertheless, current treatment strategies are limited, particularly among athletes, that are often tempted to return prematurely to play, with an inadequate recovery and high risk of reinjury [5]. 

In this scenario, HA injections seem a reliable option for the management of this disease. HA administration has demonstrated anti-inflammatory, proliferative, repairing and analgesic effects during tendinopathy, playing a role also on stiffness and tone of the tendon, identified as markers of this disease [41]. Moreover, HA seems to promote a significant and prompt clinical and functional improvements that encourage its use in athletes. 

During sports practice, particularly in competitive one and among the in-season athletes, chronic and recurrent tendinopathies are commonly reported, thus implicating a troubling return to sport (RTS), in terms of “decision-making process of returning an injured or ill athlete to practice or competition” and “according to the sport […] and the level of participation […] that the athlete aims to return to” [44]. However, this key outcome has not been investigated in studies about the HA use in sport-related tendinopathies. This is probably due to the absence of a clear consensus on the definition and criteria of RTS.

In a recent systematic review, Habets et al. defined 8 criteria to be filled, used to support the RTS decision after Achilles tendinopathy, commonly observed among elite soccer players with short recovery periods [45]. In our opinion, these criteria (Table 2) might be adaptable to both upper and lower limb tendinopathies that occurred in athletes. Taking into account all the biological actions modulated by HA on the tendon, HA injections might influence several aspects of the RTS considered by these criteria: level of pain and functional recovery as well as muscular strenght, range of motion, endurance, and anatomical properties of tendon may be positively affect by HA, although well-designed studies are needed to provide evidence for this hypothesis.

Finally, available literature suggests that HA injection might be safe and well-tolerated procedure with no serious adverse events reported [14,36,40,41].

This is the first narrative review that explored the role of HA in sport-related tendinopathies; however, the main limitation is the lack of a systematic approach. It would be interesting to produce systematic reviews and meta-analysis on the use of HA for each tendon injury, addressing an unmet topic for athletic population.

## 4. Conclusions

Tendinopathy is a common sport-related injury, due to overuse and repetitive loading, with a progressive accumulation of damage in tendon tissue.

Emerging therapeutic options include the use of HA, although current recommendations still not consider this intervention among the approved treatment strategies for the management of tendinopathies, also in athletes. 

Further research with more rigorous methods, adequate sample size, long-term follow up and instrumental assessment of tendon damage are needed to improve the biological and clinical knowledge about HA as a viable therapeutic option in the management of sport-related tendinopathies.

## Figures and Tables

**Table 1:** Characteristics of the clinical studies on the HA use in tendinopathy.

Author, Year	Study Design	Disease	Sample Size: Total; Group	Intervention	Outcome	Follow-Up	Main Results
Meloni, 2008	Open-labeled prospective study	Supraspinatus tendinosis	56 patients; LMW HA (28) Saline (28)	LMW HA group: 20 mg of LMW HA, with 2 mL of 1% lidocaine and 2 mL of 0.9% sodium chloride solution. Saline group: 4 mL of 0.9% sodium chloride solution, with 2 mL of 1% lidocaine. Timing: Both groups received 5 injections over the superior tendon surface (1-week interval)	Shoulder disability, ROM, VAS	1, 3, 6, 12 months	In the LMW HA group, shoulder disability and pain improved for 25/28 patients at 1 and 3 months follow-up, 19/28 at 6 and 12 months follow-up; in the Saline group, shoulder disability and pain improved for 0/28 patients. In both groups ROM degrees were unchanged.
Petrella, 2010	Prospective randomized clinical trial	Lateral epicondylitis	331 racquet sport athletes; HA (165) Saline (166)	HA group: 1.2 mL HA (1% HA in a phosphate buffered saline contained in a prefilled syringe); Saline group: 1.2 mL saline placebo. Timing: Both groups received 2 injections into the subcutaneous tissue and muscle 1 cm from the lateral epicondyle (1-week interval)	VAS-pain at rest and after grip testing	14, 30, 90, 356 days	In the HA group, VAS-pain at rest and after grip testing were significantly better compared to Saline group from day 30 to each follow-up.
Muneta, 2012	Retrospective study	Patellar tendinopathy	50 athletes	25 mg of hyaluronan (molecular weight 900,000 Da) in 2.5 mL (superpurified hyaluronate, Seikagaku Kogyo Co. Ltd., Tokyo, Japan) at the proximal interface between the posterior surface of the patellar tendon and the infrapatellar fat pad	Effectiveness *, safety	25.7 months	From January 1999 to December 2006, 135 injections were performed with an average of 2 injections per case. 54% of the participants return to full athletic activities, while 40% were able to continue to participate in previous sporting activities. The rate of side effects from HA has been reported to be as low as 0.57%
Merolla, 2013	Prospective non-randomized comparative study	RC tendinopathy	48 patients; LMW HA (25) Physiotherapy (23)	LMW HA group: 12 mg/1.2 mL of STABHATM (Soft Tissue Adapted Biocompatible Hyaluronic Acid; SportVisTM, MDT Int’l SA, Switzerland) Physiotherapy group: active shoulder mobilization, soft tissue stretching and humeral head positioner and propeller muscles strengthening. Timing: LMW HA group received 2 injections over the superior tendon surface (2-week interval); physiotherapy group underwent 30 days of rehabilitation (3 sessions every week).	VAS, OSS, CMS, PGA	2, 4, 12, 24 weeks	In the LMW HA group, a statistically significant reduction of pain and improvement of both CMS and OSS were found from baseline to the week 2, 4, and 12 (*p* < 0.05); in the Physiotherapy group, a statistically significant reduction of pain and improvement of both CMS and OSS were found at week 2 (*p* < 0.05) but not at week 4, 12 and 24 (*p* > 0.05). PGA scores showed good patient compliance with no serious adverse events registered.
Tosun, 2015	Prospective randomized clinical trial	Lateral epicondylitis	57 patients; HA + CS (25) Triamcinolone (32)	HA + CS group: 1 mL of an HA + CS combination (800 mg hyaluronate combined with 1 g chondroitin sulfate/50 mL; Ialuril, IBSA Farmaceutici Italia S.r.l, Italy) + 0.6 mL of prilocaine HCl (Citanest, AstraZeneca PLC, London, UK). Triamcinolone group: 1 mL of triamcinolone acetonide (40 mg/mL; Kenacort-A Retard, Bristol Meyers Squibb Pharmaceuticals, New York, NY, USA) + 0.6 mL of prilocaine HCl (Citanest). Timing: Both groups received one injection over an area under the external origin, immediately anterior and distal to the lateral epicondyle	PRTEE questionnaire and MCID and percentage changes in the PRTEE	3, 6 months	Both groups improved mean pain and function scores at 3 and 6 months. HA + CS group showed a statistically significantly better mean function score and mean pain and function scores at 3 and 6 months, respectively, compared to triamcinolone group. HA + CS group showed clinically significant improvements at 3 and 6 months compared with triamcinolone group.
Lynen, 2016	Multinational, prospective, randomized controlled, blinded-observer trial.	Achilles’ tendinopathy	59 patients; HA (29) ESWT (30)	HA group: 40 mg/2 mL + 10 mg mannitol, OSTENIL^®^ TENDONa ESWT group: piezo-electric ESWT device (PiezoSon 100 plusb) with standardized parameters (10 mm penetration depth, 94°aperture angle, 4 Hz pulse frequency, 1500 pulses per application) with an intensity levels set to 14 and 15 Timing: HA group received 2 injections peritendinously at the midportion of Achilles’ tendon (1-week interval); ESWT group: 3 sessions at weekly intervals.	VAS, VISA-A, safety	1, 2 **, 4 ***, 12 ***, 24 *** weeks	In the HA group, percent change in pain (VAS) and VISA-A scores were statistically significant at 1, 3, 6 months compared to ESWT. No serious adverse events was reported.
Gervasi, 2021	Pilot study	Achilles’ tendinopathy	8 male runners	Three injections of 2–1000 kDa HA in 2 mL, (RegenFlex T&M, Regenyal Laboratories SRL, Italy), one each 15-day	Pain (Likert-scale 0–5), biochemical markers, MVIC, viscoelastic properties such as tone [frequency of oscillation (Hz)] and siffness) through handheld myotonometer, safety	0, 15, 30, 45 days	Pain, MVIC, tone, and stiffness were significantly different between healthy and injured tendon during all over time. A reduction of IL1-β and MMP-3 level and pain plus an amelioration of tone, stiffness and MVIC was reached in the injured tendon among the visits. No adverse event was reported.

Abbreviations: LMW HA (low molecular weight hyaluronic acid); ROM (range of motion); VAS (visual analogue scale); RC (rotator cuff); STABHATM (Soft Tissue Adapted Biocompatible Hyaluronic Acid); OSS (Oxford Shoulder Score); CMS (Constant–Murley Score); PGA (Patient Global Assessment); CS (chondroitin sulfate); PRTEE (Patient-Rated Tennis Elbow Evaluation) questionnaire; MCID (Minimum Clinically Important Difference); ESWT (extracorporeal shock wave therapy); VISA-A score (Victorian Institute of Sports Assessment Achilles questionnaire); MVIC (maximal voluntary isometric contraction). * scored as four grades on the basis of subjective pain felt during sporting activities combined with patient’s ability to participate in athletic activities: “excellent”—fully return to their previous sporting level, “good”—participate in the sporting activities that they did previously, albeit with some limitations, “fair”—some improvement after the injection but still difficult to return to the previous sporting level, “poor”—unable to return to the previous sporting level with or without transient improvement) ** for the ESWT group. *** after the last treatment administration.

**Table 2:** Proposal of RTS criteria after Achilles tendinopathy [45].

level of pain
level of functional recovery
muscular strength
range of motion
endurance
medical advice
psychosocial factors
anatomical/physiological properties

## Data Availability

Not applicable.
